# Differences in gene expression between high‐grade dysplasia and invasive HPV^+^ and HPV^−^ tonsillar and base of tongue cancer

**DOI:** 10.1002/cam4.2450

**Published:** 2019-08-27

**Authors:** Linnea Haeggblom, Andreas Ährlund‐Richter, Leila Mirzaie, Pedro Farrajota Neves da Silva, Ramona G. Ursu, Torbjörn Ramqvist, Anders Näsman

**Affiliations:** ^1^ Department of Oncology‐Pathology Karolinska Institute Stockholm Sweden; ^2^ Department of Clinical Pathology Karolinska University Hospital Stockholm Sweden; ^3^ Department of Microbiology University of Medicine and Pharmacy Grigore T. Popa Iasi Iasi Romania

**Keywords:** base of tongue cancer, cancer in situ, cancer progression, high‐grade dysplasia, human papillomavirus, invasive carcinoma, oropharyngeal cancer, premalignant stages, tonsillar cancer

## Abstract

**Background:**

Human papillomavirus (HPV) is a causative agent for tonsillar and base of tongue squamous cell carcinoma (TSCC/BOTSCC), as well as for cervical cancer. Premalignant stages in cervical cancer have been studied extensively, while little is known about premalignant stages in TSCC/BOTSCC and the role of HPV. Here we analyzed differences in gene and protein expression between high‐grade dysplasia and invasive cancer in both HPV‐positive (HPV^+^) and HPV‐negative (HPV^−^) TSCC/BOTSCC.

**Methods:**

High‐grade dysplasia and invasive carcinoma were laser microdissected from HPV^+^ and HPV^−^ TSCC/BOTSCC tumor sections. Differential gene expression was studied utilizing nanoString RNA‐panels and genes of interest were validated on the protein level by immunohistochemistry.

**Results:**

Forty genes in the HPV^+^ tumors showed significantly different expression between high‐grade dysplasia and invasive cancer and 33 genes in the HPV^−^ tumors. Five out of the nine most significant pathways showed similar increased activity in invasive cancer as compared to high‐grade dysplasia in both HPV^+^ and HPV^−^ tumors. Lastly, significant differences in protein expression was confirmed for SPARC, psoriasin, type I collagen and galectin‐1 in both HPV^+^ and HPV^−^ tumors.

**Conclusions:**

This is to our knowledge the first study disclosing differences and similarities in gene expression between dysplastic and invasive HPV^+^ and HPV^−^ TSCC/BOTSCC.

## Graphical abstract

It is debated if high‐grade dysplasia exists in HPV+ TSCC/BOTSCC, there is also a need for pathological invasion markers in HNSCC. Here we present similarities between HPV+ and HPV‐ high‐grade dysplasia, suggesting HPV+ high‐grade dysplasia is an entity. We also identify possible invasion markers in HNSCC.

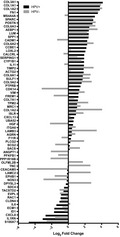

**Abbreviations**
AJCC: American joint committee on cancer
BOTSCC: base of tongue squamous cell carcinoma
CIN: cervical intraepithelial neoplasia
DCIS: ductal carcinoma in situ
ECM: extracellular matirx
FFPE: formalin fixed paraffin embedded
HNSCC: head and neck squamous cell carcinoma
HPV: human papillomavirus
HPV^−^: human papillomavirus negative
HPV^+^: human papillomavirus positive
HSIL: high‐grade squamous intraepithelial lesions
IHC: immunohistochemistry
LCM: laser capture microdissection
LSIL: low‐grade squamous intraepithelial lesions
OPSCC: oropharyngeal squamous cell carcinoma
p16: p16^INK4a^ tumor suppressor protein
SPARC: secreted protein acidic and rich in cysteine
TIL: tumor infiltrating lymphocyte
TSCC: tonsillar squamous cell carcinoma

## 1 BACKGROUND

Previous studies by us and others have demonstrated an epidemic increase in the incidence of tonsillar and base of tongue squamous cell carcinoma (TSCC and BOTSCC), and this has been attributed to an increase in human papillomavirus (HPV) infection.^1^, ^2^, ^3^, ^4^, ^5^, ^6^, ^7^, ^8^ Moreover, patients with HPV positive (HPV^+^) TSCC and BOTSCC are generally younger and do not usually present with the classical head and neck squamous cell carcinoma (HNSCC) risk factors, for example, smoking and alcohol abuse.^9^, ^10^ However, despite an epidemic increase in a younger patient population, no screening methods, in line with those for cervical cancer, have been developed.

The absolute majority of all carcinomas develop in a similar manner, starting with dysplasia followed by high‐grade dysplasia/cancer in situ, which eventually progresses into an invasive cancer and thereafter becomes metastatic. High‐grade dysplasia/cancer in situ refers to the earliest stage of cancer when the cancerous growth is still contained by the basal membrane and has not invaded the surrounding tissues or spread to other organs in the body.^11^ In cervical carcinoma, the premalignant and malignant stages are very well characterized and divided into low‐grade squamous intraepithelial lesions (LSIL) and high‐grade squamous intraepithelial lesions (HSIL) (former cervical intraepithelial neoplasia [CIN] 1‐3 grades) and invasive cancer. Analogous stages are also found in other HPV‐associated anogenital carcinomas.^12^ A similar, yet less studied, scenario is also observed in the epithelium coating the tonsils and the base of tongue, where occasionally premalignant lesions are found close by the invasive tumor. However, it has been debated if pure premalignant lesions occur in HPV^+^ TSCC or BOTSCC.^13^, ^14^, ^15^, ^16^

Increased knowledge of genes involved in tumor invasion mechanisms and identification of new progression markers could potentially in the future improve and personalize treatment and/or facilitate development of new strategies for early detection and preventative measures.

Due to its rarity, no studies have, to our knowledge, been performed on differences between premalignant and invasive lesions in HPV^+^ TSCC and BOTSCC. Instead, a handful of studies have been performed on premalignant changes in HPV^+^ cervical carcinomas. In two independent studies by Gius et al and den Boon et al, differences in gene expression in the different stages of premalignant lesions (CIN1‐3) leading up to cervical cancer were examined, and both studies show specific changes displayed from stage‐to‐stage.^17^, ^18^ However, no comparisons between HPV^+^ and HPV negative (HPV^−^) precancerous and invasive tumors were possible in these studies, since >99% of all cervical carcinomas are HPV^+^.^19^, ^20^

Moreover, it is imperative for the patient treatment and prognosis to distinguish dysplasia/cancer in situ from an invasive carcinoma. Whearas an invasive tumour diagnosis most often will result in heavy oncological treatment and/or radical surgery, a diagnosis of cancer in situ/severe dysplasia diagnosis may lead to surgical extirpation, but not with extensive margins, and only clinical follow‐up. Normally, the distinction between premalignant and invasive disease is unproblematic, but there are cases, especially in head neck cancer pathology when the diagnosis is vague. Therefore, some invasion markers, for example, Laminin‐5,^21^ have been proposed, but these markers have all very poor sensitivity and specificity in the head and neck area. Therefore, there is an urgent need for new clinical invasion markers in head and neck pathology, for better and safer diagnosis of invasive disease.

In this study we aimed, for the first time, to examine and better understand differences between HPV^+^ and HPV^−^ preinvasive TSCC and BOTSCC lesions and invasive tumors by laser capture microdissection (LCM) of the malignant epithelial cells followed by gene analysis utilizing the PanCancer Progression Panel (NanoString). In addition, we also aspired to identify usable clinical invasion markers by visual verification of obtained RNA data on protein level by immunohistochemistry (IHC).

## 2 METHODS

### 2.1 Patients and tumor samples

In this study 24 TSCC and BOTSCC (ICD‐10: C09.0, C09.1, C09.8, C09.9, C01.9) biopsies were selected from a larger consecutive cohort diagnosed at Karolinska university hospital 2007‐2015.^5^, ^22^ All biopsies, previously tested for both presence of HPV DNA and p16^INK4a^ (p16) expression,^5^, ^22^ were histologically evaluated and selected for containing both high‐grade dysplasia/cancer in situ and invasive carcinoma within the same formalin fixed paraffin embedded (FFPE) section. Thirteen HPV driven tumors, being both HPV DNA positive and p16 positive (HPV^+^p16^+^) were identified from the cohort and 11 HPV DNA negative and p16 negative (HPV^−^p16^−^) tumors, thus driven by other factors, were selected. From these 24 samples, the six HPV^+^ and HPV^−^ patient samples with highest quality and satisfactory amount of RNA were used in the RNA expression assay (please see below). Differentially expressed genes were then validated in all 24 samples by IHC. For more details about the study subjects see Supplementary Tables S1 and S2. The study was performed according to approval 2009/1278‐31/4 from the Regional Ethics Committee, Karolinska Institutet.

### 2.2 Tumor selection and microdissection

**Figure 1:**
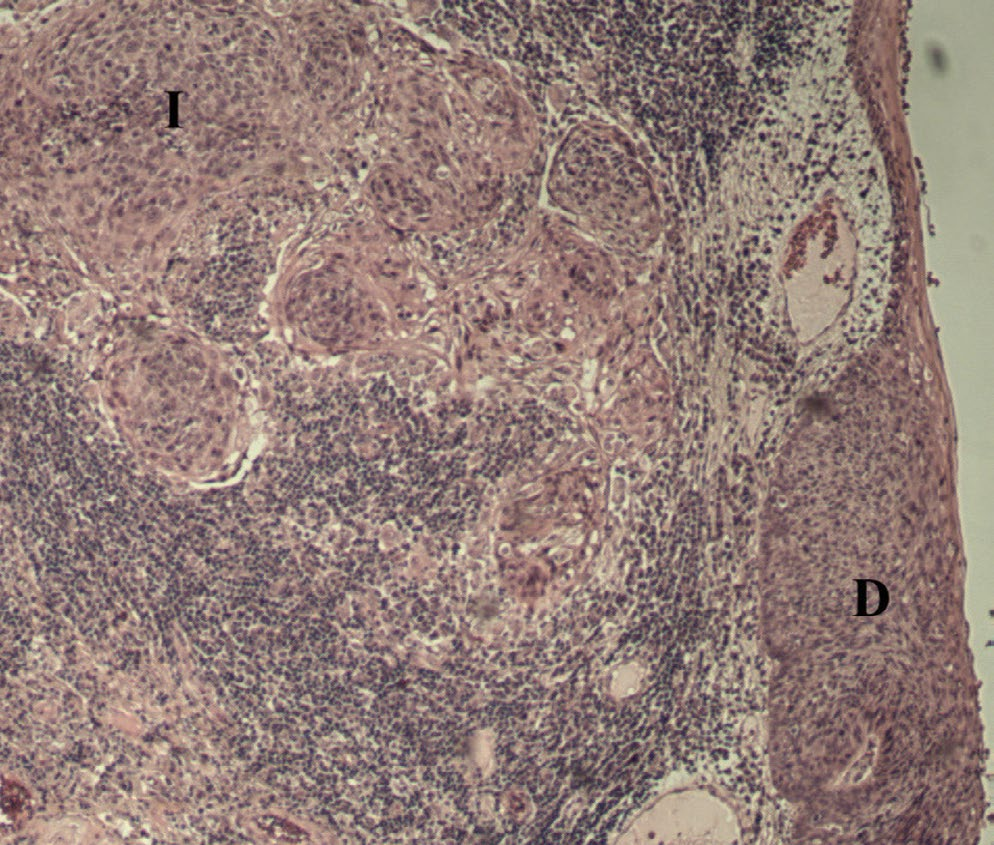
Histological representation of contained high‐grade dysplasia/cancer in situ (D) and invasive carcinoma (I)

Hematoxylin/eosin stained FFPE tumor sections were histologically examined by three independent researchers (LH, PFNS, and AN) and selected for containing sufficient amounts of high‐grade dysplasia/cancer in situ as well as invasive cancer (Figure 1). FFPE biopsies with distinct differences for dysplasia and invasive carcinoma were sectioned (six 5 μm‐sections per sample) by microtome with RNase‐free water and mounted on Membrane Slides PEN‐Membrane 2.0 µm (No.11505158) (Leica Microsystems AB). Tissue sections were deparaffinized for 2 × 15 minutes in fresh xylene followed by rehydration in decreasing percentages of fresh ethanol (5 minutes each in 100%, 95%, 70%, 50%, 0% ethanol) and thereafter stained with fresh hematoxylin for 30 seconds. On a Leica LMD 7000 microscope (Leica Microsystems AB), using the Laser Microdissection System (version 7.6.5684) the dysplastic and invasive carcinoma areas of the six replicate tumor sections were laser micro dissected and collected separately in microcentrifuge tubes containing PKD buffer from the RNeasy FFPE kit (Qiagen).

### 2.3 RNA extraction and multiplex gene expression analysis

Samples were kept on ice whenever possible and RNA was extracted immediately upon laser microdissection to ensure best RNA quality, using the RNeasy FFPE kit (Qiagen) according to manufacturer's instructions. RNA concentration was measured on Qubit 4 Fluorometer (Thermo Fisher Scientific) using the Qubit™ RNA HS Assay Kit (Thermo Fisher Scientific). Twelve samples (six HPV^+^p16^+^ tumors and six HPV^−^p16^−^ tumors) with sufficient RNA concentrations (>2 ng/μL) were selected for the multiplex gene expression assay. Due to low RNA concentrations from FFPE material, the nCounter Low RNA Input Amplification Kit (NanoString Technologies) was used for the cDNA conversion and multiplex target enrichment steps. Thereafter the hybridization reactions were set up using provided master kit and progression primers needed for the nCounter PanCancer Progression Panel (NanoString Technologies). The gene expression assay was performed according to manufacturer's instructions utilizing the nCounter^®^ Sample Prep Station with FLEX configuration (NanoString Technologies) as well as the nCounter^®^ Digital Analyzer 5s (NanoString Technologies) for reading the samples. For data analysis the nSolver Analysis Software (version 4.0) was used, including the nCounter Advanced Analysis add‐on software (version 2.0.115). More specifically, fold changes and *P*‐values were calculated using nCounter default settings. Pathway scores were calculated using measurements of genes included in 34 different pathways, using the nCounter Advanced Analysis Software.^23^

### 2.4 Immunohistochemistry evaluation of differentially expressed genes

Six of the top differentially expressed genes between high‐grade dysplasia/cancer in situ and invasive carcinoma, identified from the multiplex gene expression analysis, were further evaluated for protein expression by IHC. FFPE sections from the 24 TSCC/BOTSCC biopsies were stained by IHC with six different antibodies as per standard protocol. Further details about antibodies and methodologies are presented in Supplementary Table S3. Stained tissue sections were histologically evaluated by light microscopy by three trained scientists (LH, AN, LM) as described separately for each antibody in Supplementary Table S3. Evaluation scores are presented in Supplementary Table S4.

### 2.5 Statistical analysis

Nanostring data and pathway analysis are described above. Differences in protein expression were assessed with the Wilcoxon signed rank‐test. Two sided *P*‐values were reported.

## 3 RESULTS

### 3.1 Gene expression and pathway analysis of high‐grade dysplasia and invasive TSCC and BOTSCC

**Figure 2:**
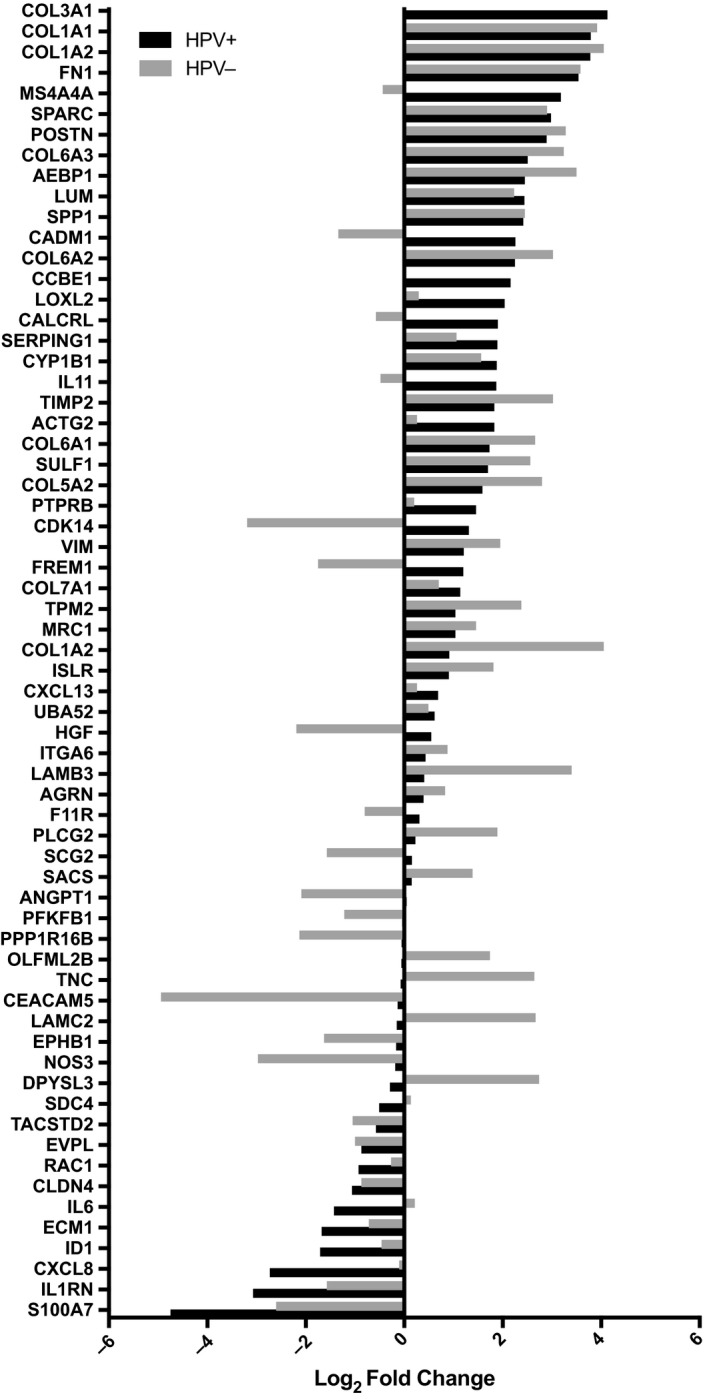
Log_2_ fold change difference between high‐grade dysplasia and invasive carcinoma comparing statistically significant differences (*P* < .05) in mRNA expression in the HPV^+^ and HPV^−^ samples separately. All genes showing a positive fold change value have a higher expression in invasive carcinoma compared to high‐grade dysplasia, and genes showing a negative fold change value have a lower expression in invasive carcinoma compared to high‐grade dysplasia

Gene expression was measured in six HPV^+^ and six HPV^−^ TSCC and BOTSCC samples from a panel of 770 cancer progression related genes (PanCancer Progression Panel, NanoString). In total, 40 genes in the HPV^+^ tumors and 33 genes in the HPV^−^ tumors showed differential expression (*P* < .05), comparing high‐grade dysplastic epithelium and invasive cancer. Significant genes are presented in Figure 2 for both HPV^+^ and HPV^−^ tumors. Of these genes, 10 were common for both HPV^+^ and HPV^−^ tumors. More specifically, the genes *COL3A1, COL1A1, COL1A2, SPARC, COL6A3, AEBP1, COL6A2,* and *VIM* were significantly up‐regulated, whereas *S100A7* and *TACSTD2* were significantly down‐regulated, in invasive cancer as compared to the dysplastic epithelium (Figure 2).

Consequently, 30 genes differed between high‐grade dysplasia vs. invasive cancer uniquely for HPV^+^ samples. The following five genes, *FN1, MS4A4A, POSTN, LUM,* and *SPP1,* were the most significantly up‐regulated, in invasive cancer as compared to the dysplastic epithelium. Whereas *IL1RN, CXCL8, ID1, ECM1,* and *IL6*, were the five most significantly down‐regulated genes in invasive cancer as compared to the dysplastic epithelium. For more detailed data see Figure 2.

Exclusively for HPV^−^ cases 23 genes differed between high‐grade dysplasia and invasive cancer. The following five genes, *LAMB3, TIMP2, COL5A2, DPYSL3,* and *LAMC2,* were the most significantly up‐regulated, in invasive cancer as compared to the dysplastic epithelium. Whereas *CEACAM5, CDK14, NOS3, HGF,* and *PPP1R16B,* were the five most significantly down‐regulated genes in invasive cancer as compared to the dysplastic epithelium (Figure 2).

**Figure 3:**
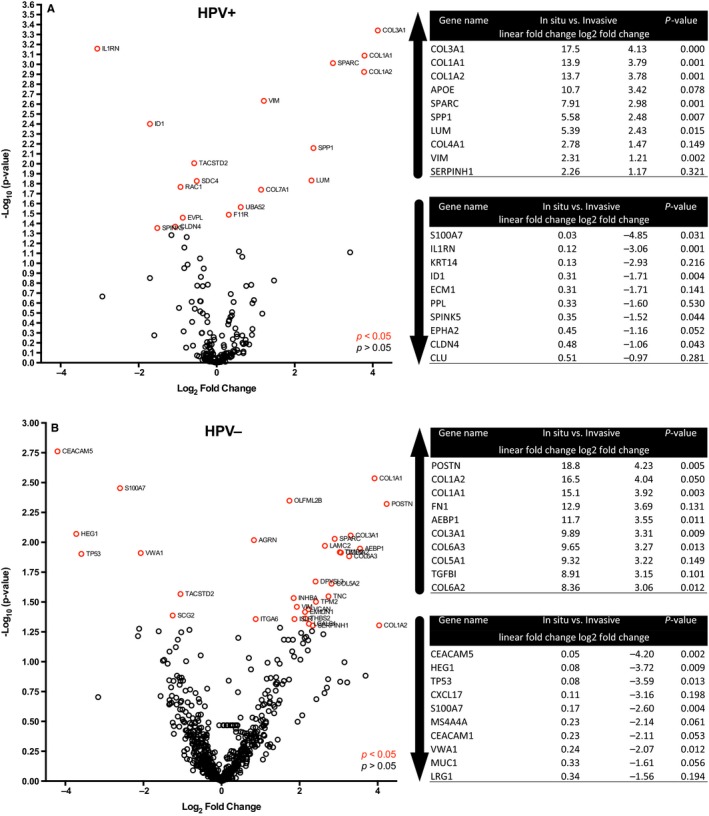
Gene expression in high‐grade dysplasia and invasive carcinoma in HPV^+^ and HPV^−^ tonsillar and base of tongue squamous cell carcinoma separately. (A) Volcano plot showing differentially expressed genes between high‐grade dysplasia and invasive carcinoma by log_2_ fold change (*x*‐axis) and −log_10_ of *P*‐value (*y*‐axis) in HPV^+^ tonsillar and base of tongue squamous cell carcinoma cases. The top 10 genes with increased and decreased linear fold change as well as log_2_ fold change expression between high‐grade dysplasia and invasive carcinoma are together with *P*‐values presented in the tables to the right. (B) Same as in (A), here however with HPV^−^ tonsillar and base of tongue squamous cell carcinoma cases

Upon further evaluation of the above mentioned genes, a more stringent cut‐off was applied in order to exclude samples with a low count value, resulting in that 19 genes in the HPV^+^ samples (Figure 3A) and 32 in the HPV^−^ samples (Figure 3B) presented with differentially expressed genes.

**Figure 4:**
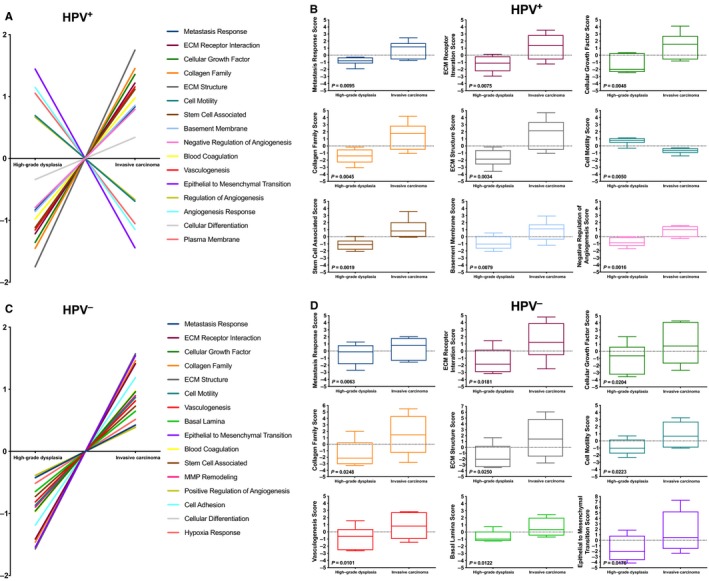
Pathway differences between high‐grade dysplasia and invasive carcinoma. Individual pathway scores for high‐grade dysplasia and invasive carcinoma are shown for (A) and (C) respectively in HPV^+^ and HPV^−^ tonsillar and base of tongue squamous cell carcinoma respectively. Boxplots for individual pathway scores for the nine most statistically significant different pathways are illustrated in (B) and (D) respectively for HPV^+^ and HPV^−^ tonsillar and base of tongue squamous cell carcinoma

To characterize the effect of altered gene expression on various cancer progression pathways, the pathway analysis tool provided in the nSolver Advanced Analysis Software was utilized. This tool condenses each sample's gene expression profile to calculate a pathway score using a first principal component analysis.^23^ Notably, among the nine most significant pathways, there was a similar increased activity in invasive cancer as compared to high‐grade dysplasia in both HPV^+^ and HPV^−^ tumors in five pathways: metastasis response, extracellular matrix (ECM) receptor interaction, cellular growth factor, collagen family, and ECM structure (Figure 4).

**Figure 5:**
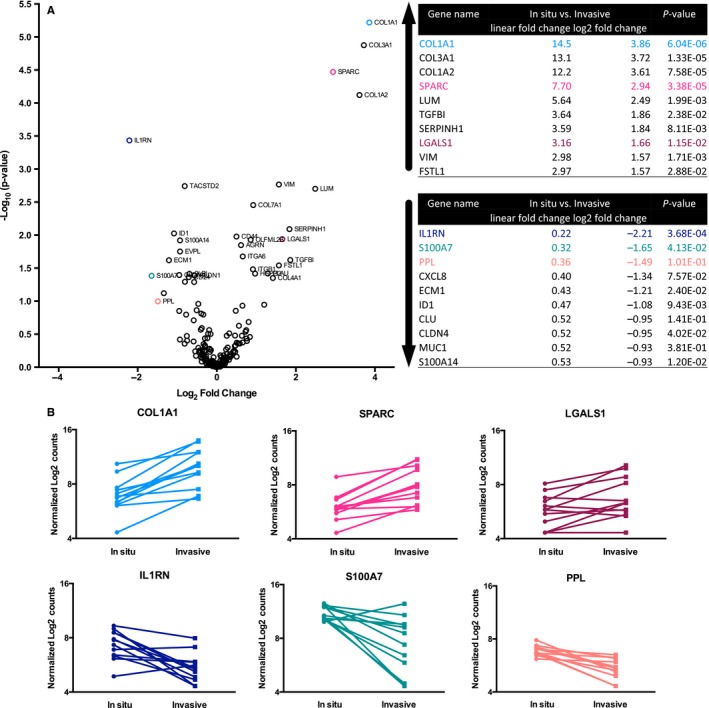
Gene expression in high‐grade dysplasia and invasive carcinoma of both HPV^+^ and HPV^−^ tonsillar and base of tongue squamous cell carcinoma samples combined, adjusted for HPV^+^ status. (A) Volcano plot showing differentially expressed genes between high‐grade dysplasia and invasive carcinoma by log_2_ fold change (*x*‐axis) and −log_10_ of *P*‐value (*y*‐axis) in HPV^+^ and HPV^−^ tonsillar and base of tongue squamous cell carcinoma cases. The top 10 genes with increased and decreased linear as well as log_2_ fold change expression between high‐grade dysplasia and invasive carcinoma are together with *P*‐values presented in the tables to the right. Genes marked with a color were selected for immunohistochemical protein analysis. (B) Graphs of selected genes showing normalized log_2_ counts for tonsillar and base of tongue dysplastic lesions and invasive carcinoma (HPV^+^ and HPV^−^) for each individual sample

In the search of new clinical invasion biomarkers in head and neck cancer, and because of low sample number, the HPV^+^ and HPV^−^ groups were combined to investigate gene expression, adjusted for HPV status (Figure 5A). In invasive cancer vs. high‐grade dysplasia, *COL1A1, COL3A1, COL1A2,* and *SPARC* were the most up‐regulated genes, while *IL1RN*, *S100A7,* and *PPL* were the most down‐regulated genes (Figure 5A). After analyzing the fold‐change values for mRNA expression, *P*‐values and published gene functions, six genes: *COL1A1, SPARC, LGALS1, IL1RN, S100A7,* and *PPL* (Figure 5B), were chosen as potential pathological biomarker candidates for further evaluation by IHC for protein expression.

### 3.2 Protein expression in high‐grade dysplasia and invasive TSCC and BOTSCC

Based on the results from the gene expression assay for the combined cohort of HPV^+^ and HPV^−^ TSCC, six proteins (type I collagen (*COL1A1*), SPARC (*SPARC*), galectin‐1 (*LGALS1*), IL1‐RA (*IL1RN*), psoriasin (*S100A7*) and periplakin (*PPL*)) were selected for evaluation by IHC as potential invasion marker candidates. The transcripts of these six proteins showed with few exceptions either an increase (type 1 collagen, SPARC and galectin‐1) or decrease (IL1‐RA, psoriasin and periplakin) between high‐grade dysplasia and invasive cancer (Figure 5B).

**Figure 6:**
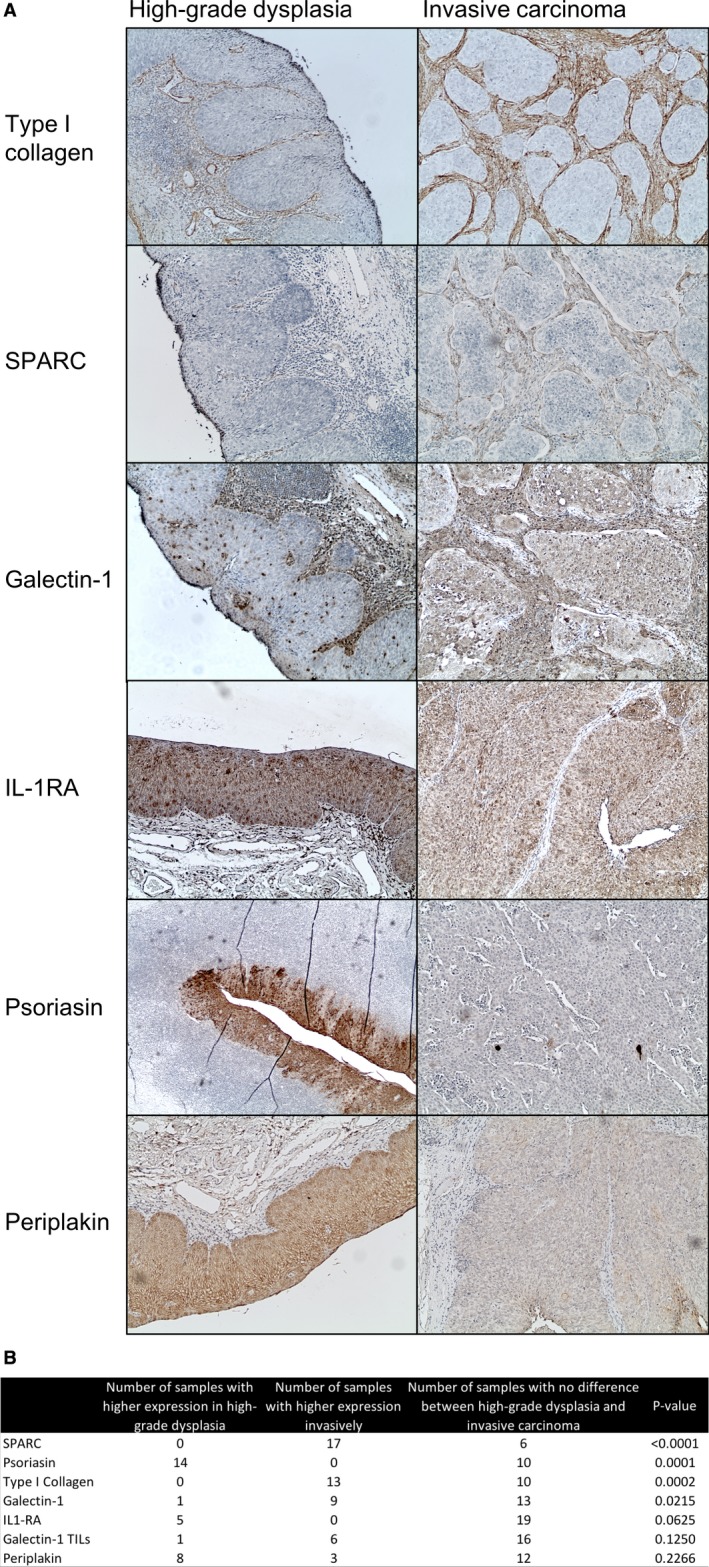
(A) Examples of immunohistochemistry stainings of type I collagen, SPARC, galectin‐1, IL‐1RA, psoriasin, and periplakin, in high‐grade dysplasia/carcinoma in situ an in invasive carcinoma. Expression of type I collagen and SPARC is higher in the tissue surrounding invasive carcinoma, and galectin‐1 has a higher expression in invasive carcinoma, whereas IL‐1RA, psoriasin, and periplakin show a higher expression in high‐grade dysplasia compared to invasive carcinoma. (B) Summary of immunohistochemical protein evaluation scores and *P*‐values for selected proteins, HPV^+^, and HPV^−^ tumors combined

Protein expression of the above genes was therefore evaluated on the extended cohort with 24 different FFPE tumor sections, but due to lack of sufficient tumor material one sample was excluded for type I collagen, SPARC, galectin‐1, and periplakin. Details of the staining evaluation and scores are shown in Figure 6 and Supplementary Tables S3 and S4 respectively, and examples of the stainings are presented in Figure 6.

Neither type I collagen nor SPARC were found to be expressed at all in dysplastic epithelium or in invasive carcinomas. However, type I collagen was often expressed in the immediate ECM surrounding the cancer, but mostly to a lesser extent around high‐grade dysplasia as compared to invasive tumor areas (*P* = .0002) (Figure 6). Similarly, SPARC was often expressed in the peritumoral stroma in peritumoral fibroblasts, mostly with absent or little expression around dysplasia, whereas higher expression was found around 17/23 invasive cancer areas, while 6/23 samples showed no difference in expression (*P* < .0001) (Figure 6).

Galectin‐1 was mainly highly expressed in most tumors, with similar expression in high‐grade dysplastic epithelium and invasive tumor areas in 13/23 cases, while 9/23 cases showed higher expression in the invasive component, and 1/23 showed lower expression (*P* = .02) (Figure 6). Since galectin‐1 is variably expressed on immune cells,^24^ we also assessed tumor infiltrating lymphocytes (TILs) in dysplasia and invasive components, but no differences were observed in galectin‐1 positive TILs in the different components (Figure 6).

Notably, 14/24 tumors showed that psoriasin was highly expressed in epithelial dysplasia, while the remaining samples showed no difference in expression (*P* = 0001) (Figure 6). Periplakin was expressed very heterogeneously in both dysplasia and invasive tumor areas, often with no clear differences between dysplasia and invasive tumor, with a tendency toward having higher expression in invasive carcinoma. Likewise, IL1‐RA expression was mostly strongly expressed in cancer cells, however, a significant difference between high‐grade dysplasia and invasive cancer was not observed. (Figure 6).

## 4 DISCUSSION

In this study we report, for the first time, differences in gene expression between high‐grade dysplasia/cancer in situ and invasive HPV^+^ and HPV^−^ TSCC/BOTSCC. Some of the most significant differential mRNA expressions were found for *COL1A1*, *SPARC,* and *LGALS1* that were stronger expressed in invasive carcinoma compared to high‐grade dysplasia, and the opposite was shown for *S100A7*, which was stronger expressed in high‐grade dysplasia. These findings were also confirmed on a protein level by IHC. Moreover, we show that there are many similarities in gene expression and signal pathway activities between HPV^+^ and HPV^−^ tumors—especially in genes related to ECM, highlighting the importance of the tumor micro milieu.

As noted in the introduction, malignant invasive epithelial tumors develop from a dysplastic epithelium and detection of dysplasia has been utilized in different screening programs, for example, in cervical cancer. However, dysplasia in HPV^+^ TSCC/BOTSCC is rarely detected and pure premalignant stages have not been found, but common in HPV^−^ TSCC/BOTSCC. Some authors have even postulated that HPV^+^ premalignant phases do not exist in TSCC/BOTSCC,^16^ but have been described by others.^14^, ^25^, ^26^ Subsequently, only two studies have to our knowledge investigated HPV^+^ TSCC/BOTSCC dysplasia on a molecular level. Mooren et al, studied presence of p16INK4a expression in tonsillar dysplasia and found that p16INK4a positivity correlated to HPV status.^25^ Additionally, another study by Masterson et al confirmed increased SYPC2 expression in premalignant carcinoma in situ and invasive carcinoma vs. normal epithelium by LCM and qPCR. 26 However, in this study we aimed to examine a wider range of genes and their differential expression between dysplastic and invasive HPV^+^ and HPV^−^ TSCC/BOTSCC. Notably, numerous similarities in differential expression were here observed between HPV^+^ and HPV^−^ TSCC/BOTSCC dysplasia and invasive cancer, suggesting that HPV^+^ dysplasia in tonsils/base of tongue is a distinguishable entity from invasive HPV^+^ TSCC/BOTSCC. Therefore, it is very likely that premalignant stages of HPV^+^ TSCC/BOTSCC exist. Why such stages have not been described so far,^14^, ^15^ may be due to that tonsillar/base of tongue neoplasias are uncommon or possibly due to a more aggressive behavior of HPV^+^ dysplasia, an assumption that is not quite supported by data presented above. Hence, larger cohorts may be needed to identify premalignant phases in tonsils/base of tongue—possibly also by utilizing markers described here, such as psoriasin.

On the signaling pathway level, it is not surprising that pathways involved in tumor progression, such as metastasis response, ECM receptor interaction, ECM structure, collagen family, and cellular growth factor pathways are up‐regulated both in HPV^+^ and HPV^−^ invasive carcinoma. Interestingly, we also observed a higher stem cell associated score in invasive cancer compared to dysplasia, which was especially observed in the HPV^+^ samples while present to a lesser extent in the HPV^−^ samples. Notably, a study by Zhang et al showed that the cancer stem cell pool was much higher in HPV^+^ OPSCC as compared to HPV^−^ cancer.^27^ However, high expression of stem cell markers have been shown to be associated with a poor prognosis in HNSCC,^28^, ^29^, ^30^ yet most patients with HPV^+^ tumors in general have a very good prognosis.

On the transcript level, the collagen genes *COL1A1, COL1A2, COL3A1*, where in our study found to be the most significantly increased ones in invasive cancer as compared to dysplasia in both HPV^+^ and HPV^−^ tumors. Moreover, the proteins encoded by these genes belong to a family of proteins that strengthen and support many tissues in the body, being the most abundant part of the ECM.^31^ Their expression has however also been linked to cell proliferation and tumor progression and the correlation to prognosis in various cancer types.^32^ Thus, collagen has been suggested as a double‐edged sword in tumor progression, in one way acting as a passive barrier to resist tumor cells, in another way sending out biochemical and biophysical signals that affect, for example, cell adhesion, migration, angiogenesis, and repair systems that in cancer typically can be deregulated causing tumor progression.^33^ Another matricellular protein, SPARC (secreted protein acidic and rich in cysteine), which regulates cell‐matrix interactions and signaling pathways in cells, was also observed to increase in invasive cancer as compared to dysplasia. SPARC, which is considered to be solely produced by cancer associated fibroblasts in the tumor stroma, is suggested to regulate tumor cell growth and metastasis.^34^, ^35^ In a study by Witkiewicz et al, SPARC expression was correlated to tumor recurrence in ductal carcinoma in situ (DCIS) of the breast.^36^ Moreover, Chang et al show that in HNSCC a higher SPARC expression correlated to a higher tumor grade,^37^ but the prognostic role of SPARC expression is however still inconsistent within and between cancer types.^35^

*S100A7* encodes the protein psoriasin part of the S100 family containing calcium‐binding motifs and is an important cell mediator for, for example, cell survival and maturation and has been associated to tumor progression and survival.^38^ In a study by Liu et al, psoriasin was shown to promote invasion and survival of pancreatic cancer cells.^39^ Other studies have shown that psoriasin is more highly expressed in, for example, squamous skin cancer in situ compared to invasive squamous skin cancer,^40^ and in DCIS compared to invasive breast cancer,^41^, ^42^ which is in line with our findings. Tripathi et al have shown that psoriasin is significantly overexpressed in oral leukoplakia lesions with squamous cell hyperplasia or dysplasia as well as in HNSCC, compared to normal tissue.^43^ Moreover, they show that patients with a nuclear accumulation of psoriasin in HNSCC have a reduced disease‐free survival.^43^

*IL1RN* encodes the interleukin‐1 receptor antagonist (IL‐1RA) and is a natural inhibitor of the IL‐1 receptor. Expression of IL‐1RA protein has been suggested to be able to decrease tumor growth, metastasis, and angiogenesis in mouse models.^44^ In this study, we show that *IL1RN* gene expression is lower in invasive cancer compared to dysplastic epithelium, especially in HPV^+^ TSCC/BOTSCC. Interestingly, similar results have previously also been published in CIN3 vs. invasive cervical carcinoma.^18^

Lastly, in order to validate our data and in search for new pathological invasion biomarkers, we assessed six gene transcripts for protein expression and tissue distribution by IHC and were able to validate four out of six transcripts at the protein level. However, while the relative expression in dysplasia and invasive carcinoma often corresponded to the trend of transcriptional data, the absolute expression varied considerably between samples. Therefore, using any of these markers as markers of invasion in a clinical setting would probably need further tuning of staining interpretation.

There are some limitations in this study. First, tumor areas containing high‐grade dysplasia/carcinoma in situ were often scanty and decreased upon serial sectioning in some cases. Hence, the RNA concentration was very low after extraction. Due to low RNA concentrations and fragmented FFPE material, the NanoString platform using tumor progression RNA panel was utilized. However, using a more “global” method, such as RNAseq, would have yielded more data and a better picture of the transition between dysplasia and invasive tumor. Secondly, few patient samples were included in this study, reflecting that dysplasia is uncommonly detected in HPV^+^ TSCC/BOTSCC.

In conclusion, this is the first study to our knowledge, examining and disclosing differences in gene expression between dysplastic and invasive HPV^+^ and HPV^−^ TSCC/BOTSCC. However, larger studies are needed to distinguish pure premalignant from invasive tonsillar/base of tongue neoplasias.

## ETHICS APPROVAL AND CONSENT TO PARTICIPATE

Ethical approval and consent to participate was conducted according to ethical permission 2005/431‐31/4 from the Ethics Committee at Karolinska Institute, Stockholm, Sweden. The study was performed in accordance with the Declaration of Helsinki.

## CONFLICT OF INTEREST

The authors declare that they have no conflict of interest.

## AUTHOR CONTRIBUTIONS

L.H., A.N., and T.R. formulated the research question and came up with the study design. Sample selection and collection was done by A.N., P.F., and L.H. L.H. and A.N. performed the tissue microdissection, RNA extraction, and gene expression assay. A.Ä.R., L.M., and R.G.U. performed the IHC stainings. L.H., A.N., and L.M. evaluated the IHC stainings. L.H. and A.N. analyzed, summarized, and interpreted the data and wrote the manuscript which was revised and approved by all co‐authors.
